# Midtemperature CO_2_ Deoxygenation to CO over Oxygen Vacancies of Doped CeO_2_

**DOI:** 10.1021/acsami.4c17644

**Published:** 2025-05-01

**Authors:** Nan-Chian Chiang, Tz-Jie Ju, Yi-Cheng Wang, Tzu-Peng Lin, Jia-Han Guo, Shawn D. Lin

**Affiliations:** Department of Chemical Engineering, National Taiwan University of Science and Technology, Taipei 10617, Taiwan

**Keywords:** CO_2_, deoxygenation, oxygen vacancy, CeO_2_, dopant

## Abstract

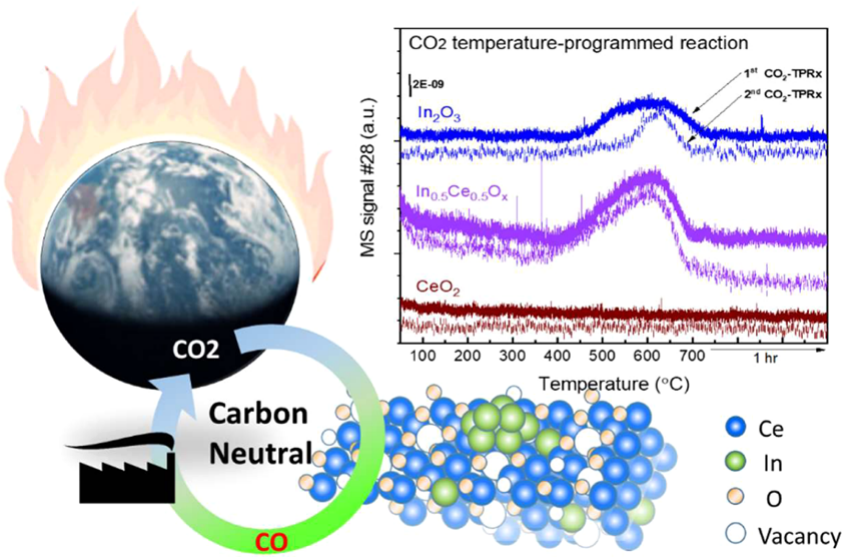

CO_2_ capture and utilization are a must for easing the global warming caused by the use of fossil fuels. Previous studies demonstrate the possibility of thermal deoxygenation of CO_2_ to CO over the vacancies of CeO_2_. This study examines the influence of the dopant to CeO_2_ on the deoxygenation of CO_2_ to CO, wherein the examined dopants include Zr, Gd, Sm, and In. Only In-doped CeO_2_ exhibits significant reactivity for CO_2_ deoxygenation in our sequential temperature-programmed reduction (TPR) and CO_2_-TPRx (temperature-programmed reaction) tests up to 700 °C. In_0.5_Ce_0.5_O_*y*_ after H_2_-TPR demonstrates a deoxygenation onset temperature as low as 400 °C and it can maintain a stable performance in cycle tests. X-ray diffraction (XRD) and X-ray photoelectron spectroscopy (XPS) analyses indicate that In exsolutes from the fluorite framework during TPR and the exsoluted In^0^ become oxidized in the subsequent CO_2_ deoxygenation reaction. XPS indicates that the redox of Ce also occurs during TPR-CO_2_-TPRx with In_0.5_Ce_0.5_O_*y*_. In_2_O_3_ by itself demonstrates a higher deoxygenation onset temperature, a lower per gram deoxygenation capacity, and a poorer stability than In_0.5_Ce_0.5_O_*y*_ under the same test conditions, while CeO_2_ is inactive. The results suggest a synergy between exsoluted In and the fluorite substrate, leading to the observed deoxygenation activity of In-doped CeO_2_.

## 1 Introduction

The mitigation of global warming is largely recognized as an important need, although global agreement on its strategy is not reached yet. It is generally agreed that the capture and utilization of CO_2_ is an inevitable approach and that the utilization of CO_2_ is a key to achieve a sustainable carbon-neutral cycle. Many studies have proposed various ways of CO_2_ utilization, using different driving forces (e.g., thermal, photochemical, and electrochemical reactions) to produce various types of target products. The formation of the CO product from CO_2_ may be an attractive straightforward approach as CO has many industrial applications.^1^ Previous works demonstrate that the CO_2_ thermal deoxygenation route can proceed over oxygen vacancies of CeO_2_ where a high reaction temperature is typically applied.^2−7^ CeO_2_ is well known to have oxygen vacancies from its partial reduction, and the redox reaction involving oxygen vacancies has been frequently proposed to contribute to catalytic activity. Furthermore, the fact that the reactivity of the mentioned oxygen vacancies can be influenced by the type of dopant and the dopant composition is generally accepted. However, how the dopant to CeO_2_ may influence its reactivity for catalyzing CO_2_ deoxygenation to CO has not been examined.

In this study, we examine the influence of four dopants, namely, Zr, Gd, Sm, and In, on CeO_2_, focusing on the reactivity toward CO_2_ deoxygenation. The selected dopants are known to form a solid oxide solution with the CeO_2_ fluorite structure and numerous examples can be found in the literature mentioning the uses of doped CeO_2_ as an effective support/catalyst for enhancing catalytic activity. In addition, Sm and Gd are popularly used dopants for achieving an enhanced oxygen conductivity, e.g., as the electrolyte of solid oxide fuel cells. Indium has recently attracted a great deal of attention for CO_2_ utilization. In_2_O_3_/ZrO_2_ is reported to effectively catalyze CO_2_ hydrogenation to methanol^8^ and In single-atom catalysts are reported to catalyze electrochemical CO_2_ reduction to various products.^9−11^

As mentioned, previous reports of the thermal CO_2_ deoxygenation over CeO_2_ largely used high reaction temperatures,^2−7^ which can pose an energy demand constraint for viable applications of such CO_2_ deoxygenation. In this study, the reactivity for midtemperature CO_2_ deoxygenation is explored related to doped CeO_2_, wherein the test conditions of both the vacancy formation temperature and the deoxygenation reaction temperature are tentatively targeted to be below 700 °C. A CO_2_ deoxygenation temperature at the midtemperature range is desirable as it can possibly lead to the integration with the midtemperature oxygen-conducting inorganic membrane to facilitate deoxygenation to achieve a sustainable CO_2_ utilization process. The results indicate that In is the only dopant in this study that can provide midtemperature reactivity for CO_2_ deoxygenation. The characterization of In-doped CeO_2_ indicates that In would exsolute from the fluorite framework during TPR and that both exsoluted In and the remaining CeO_2_-based fluorite would undergo a redox during our TPR-CO_2_-TPRx sequential tests. This suggests a synergistic effect between the exsoluted In phase and the CeO_2_-based fluorite.

## 2 Experimental Section

All of the doped CeO_2_ samples were prepared by a copreprcipitation method. Nitrate precursors, Ce(NO_3_)_3_·6H_2_O (Acros, 99.5%), Zr(NO_3_)_2_·6H_2_O, (Sigma-Aldrich, 99%), Sm(NO_3_)_2_·6H_2_O, (Acros, 99.9%), and Gd(NO_3_)_3_·6H_2_O (Sigma-Aldrich, 99.9%) were used as received. In(NO_3_)_3_·*x*H_2_O (Acros, 99.99%) was oven-dried at 100 °C to remove hydrated water before use. The nitrate precursors of the desired atomic ratio were first dissolved in deionized water, and then the precursor solution and NaOH_(aq)_ were steadily added to a flask containing NaOH_(aq)_ where the solution was constantly maintained at pH = 12 under stirring at 35 °C. After further aging, the precipitate was filtered, washed, dried at 60 °C in a vacuum oven, and then calcined at 5 °C/min to 450 °C for 5 h. ICP-AES (Angilent, 7500ce) analysis of the filtrate indicated that the precipitation was all near 100% completion.

The prepared samples were evaluated by a sequence of temperature-programmed reduction (TPR) followed by CO_2_ deoxygenation using a homemade microreactor system equipped with TCD and MS detection of the effluent stream. The TPR procedure was carried out under 10% H_2_ in a N_2_ (Linde LianHwa) flow from 50 to 700 or 900 °C at a ramp rate of 5 °C/min using TCD to detect the consumed H_2_, for which the formed H_2_O was trapped by a molecular sieve column. The CO_2_ deoxygenation was performed either by pulsing CO_2_ (10% CO_2_/He, 0.1 mL loop) into a constant He flow at isothermal conditions (700 or 500 °C) or by TPRx (temperature-programmed reaction) from 50 to 700 °C under a 5% CO_2_/Ar flow, both using MS (SRS, RGA200) as the detector. For the pulsing experiments, the per-pulse CO_2_ conversion was calculated from the fraction of CO_2_ consumed. A so-called CO_2_ deoxygenation capacity was evaluated based on the CO_2_ consumption of samples, in terms of O_stripped_ per gram, in the CO_2_ deoxygenation tests, with an assumption that one consumed CO_2_ would leave one stripped oxygen on the sample.

In-house X-ray diffraction (XRD) (Bruker D2 Phaser, with a Cu Kα source) was used to analyze the sample crystalline structure. The crystallite size was calculated by the Scherrer equation using the Warren correction of peak broadening. X-ray photoelectron spectroscopy (XPS) analysis was performed using a PerkinElmer PHI 550 spectrometer with Al Kα (1486.6 eV). The binding energy reference was C 1s at 284.8 eV. Ce M_4,5_ edge X-ray absorption near-edge spectroscopy (XANES) was performed at the National Synchrotron Radiation Research Center (NSRRC), Taiwan, with beamline #20A. Brunauer–Emmett–Teller (BET) surface area analysis was carried out with a commercial instrument (BELSORP-max). In situ diffuse reflectance infrared Fourier transform spectroscopy (DRIFTS) was carried out using a DRIFTS sampling accessory and an in situ gas cell (Herrick, Praying Mantis), using a Nicolet iS50 FTIR spectrometer equipped with a mercury–cadmium–telluride (MCT/A) detector cooled with liquid nitrogen. The spectrum was recorded by accumulating 256 scans at a resolution of 4 cm^–1^. The background spectrum was collected over the sample after TPR under Ar at specified temperatures.

## 3 Results and Discussion

### 3.1 Sequential TPR and CO_2_ Deoxygenation Tests

**Figure 1:**
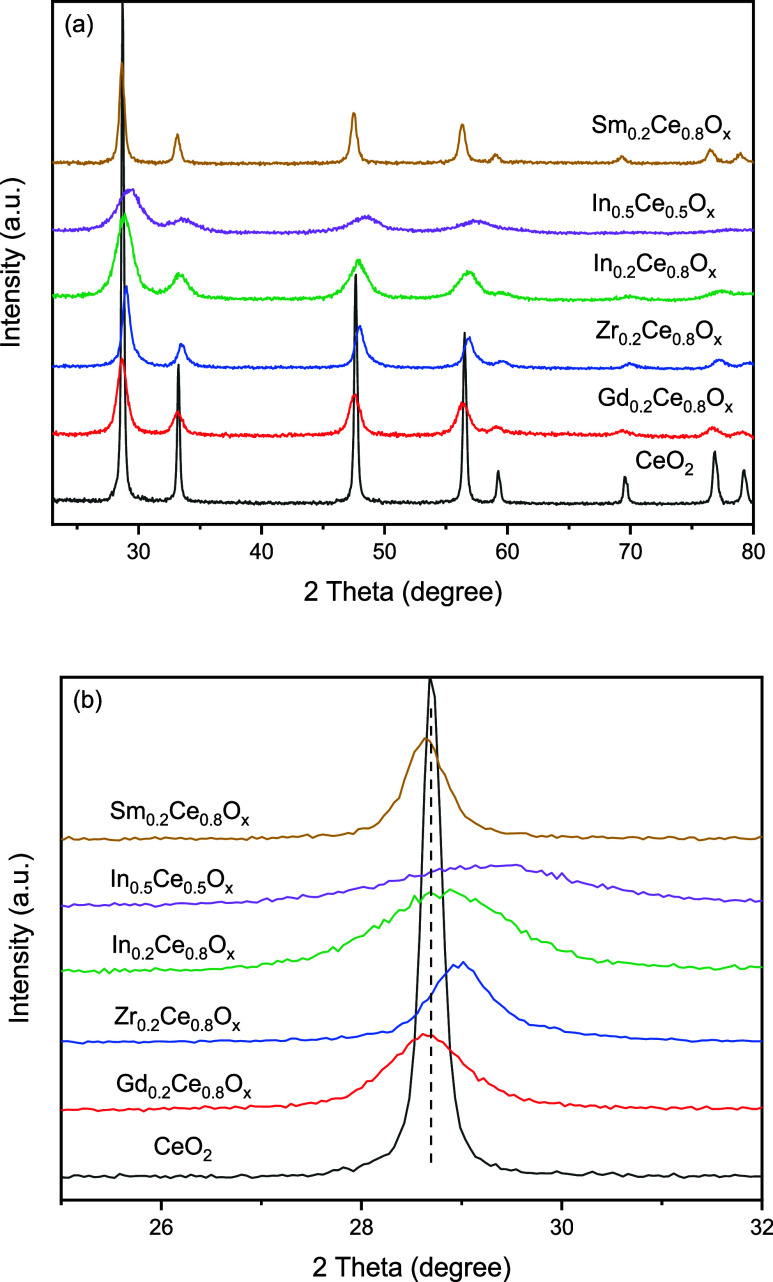
X-ray diffraction patterns of selected as-prepared M_*x*_Ce_1–*x*_O_*y*_ samples in the (a) 20–80° 2θ range and (b) expanded 2θ range revealing the shift in the fluorite(111) peak.

The dopants used to prepare M_*x*_Ce_1–*x*_O_*y*_ included Zr, Gd, Sm, and In. All of the as-prepared M_*x*_Ce_1–*x*_O_*y*_ samples exhibited only the X-ray diffraction pattern of the fluorite crystallite, as shown in Figure 1a. The absence of the impurity phase suggests that all of the dopant was well mixed into the CeO_2_ lattice, resulting in shifts in fluorite diffraction peaks qualitatively consistent with the expected lattice shrinkage or expansion corresponding to the relative size of the dopant comparing to Ce^4+^, as shown in Figure 1b. The fluorite crystallite size of the as-prepared samples was evaluated by the Scherrer equation and the peak width of CeO_2_(111), as shown in Table S1. In general, the calculated fluorite crystallite size decreased with the increasing dopant concentration.

The as-prepared samples were subjected to a sequential TPR treatment and a CO_2_ deoxygenation test, wherein the TPR treatment could generate oxygen vacancies for the subsequent deoxygenation of CO_2_. A CO_2_ deoxygenation capacity was calculated based on the consumption of CO_2_ per gram of the sample, with an assumption that one consumed CO_2_ would leave one stripped oxygen on the sample. The calculated hydrogen consumptions during the TPR of these samples are shown in Table S2. Table 1 shows the calculated CO_2_ deoxygenation capacity (as the O_stripped_ listed in Table 1) of all of the samples examined in this study, mostly with two consecutive cycles of the mentioned sequential test. Two types of CO_2_ deoxygenation tests were applied in this study, namely, an isothermal pulse input test and a TPRx test (shown as *T*_CO_2__ at 50–700 °C in Table 1). Three indexes are included in Table 1 to discuss the TPR and deoxygenation stability/reversibility, which will be elaborated later. The results listed in Table 1 show that the CO_2_ deoxygenation capacity depended not only on the dopant but also on the TPR and deoxygenation conditions. For example, a TPR treatment up to 700 °C leads to a lower CO_2_ deoxygenation capacity than a TPR treatment up to 900 °C, e.g., as observed over CeO_2_ (entries 1 and 4) and Zr_0.2_Ce_0.8_O_*y*_. However, the 700 °C TPR was preferentially examined in this study for discriminating the reactivity of different samples and also for decreasing the energy demand in future applications. From Table 1, it can be observed that only In_*x*_Ce_1–*x*_O_*y*_ samples exhibited an appreciable CO_2_ deoxygenation capacity after a TPR treatment to 700 °C.

**Table 1:** Analysis Results of the Sequential TPR-CO_2_ Deoxygenation Test over M_*x*_Ce_1–*x*_O_*y*_ Samples, Mostly for Two Consecutive Cycles

			O_stripped_ (μmol O_2_/g)				
sample	*T*_TPR_^a^ (°C)	*T*_CO_2__^b^ (°C)	first cycle	second cycle	O_2_^second^/O_2_^first^	H_2_^second^/H_2_^first^	H_2_^second^/O_2_^first^
CeO_2_	900	700	33	34	1.0	0.63	2.0
	900	500	27			0.57	2.0
	900	50–700	nil	nil		0.89	
	700	700	0.6	nil		0.31	
	700	50–700	nil	nil		0.98	
Gd_0.2_Ce_0.8_O_*y*_	900	700	20	15	0.75	0.90	1.9
	700	50–700	nil				
Gd_0.5_Ce_0.5_O_*y*_	900	700	11	9	0.82	0.72	2.1
Zr_0.2_Ce_0.8_O_*y*_	900	700	32	27	0.84	0.66	2.1
	900	500	8			0.64	
	700	700	0.5			0.56	
	700	50–700	nil				
Zr_0.5_Ce_0.5_O_*y*_	900	700	20	11	0.55	0.86	3.1
	700	50–700	nil				
Sm_0.05_Ce_0.95_O_*y*_	900	50–700	nil				
Sm_0.2_Ce_0.8_O_*y*_	900	50–700	nil				
Sm_0.5_Ce_0.5_O_*y*_	900	50–700	nil				
In_0.05_Ce_0.95_O_*y*_	900	700	43	43	1.0	0.70	2.0
	900	500	39			0.75	2.0
	900	50–700	nil	nil		0.57	
	700	700	16			0.48	2.0
	700	50–700	nil	nil		0.48	
In_0.2_Ce_0.8_O_*y*_	900	50–700	750	642	0.86	0.90	1.8
	700	50–700	774	603	0.78	0.93	1.9
In_0.5_Ce_0.5_O_*y*_	900	50–700	1727	1692	0.98	0.98	2.0
	700	50–700	1742	1731	0.99	0.98	2.0
In_2_O_3_	700	50–700	1091	794	0.73	0.80	1.9

a TPR treatment with an upper limit temperature of either 700 or 900 °C, and the calculated corresponding H_2_ consumption shown in Table S2.

b CO_2_ deoxygenation reaction carried out either isothermally with a pulse input of 5% CO_2_ or by TPRx from 50 to 700 °C under a 5% CO_2_ flow, and the calculated corresponding stripped oxygen.

**Figure 2:**
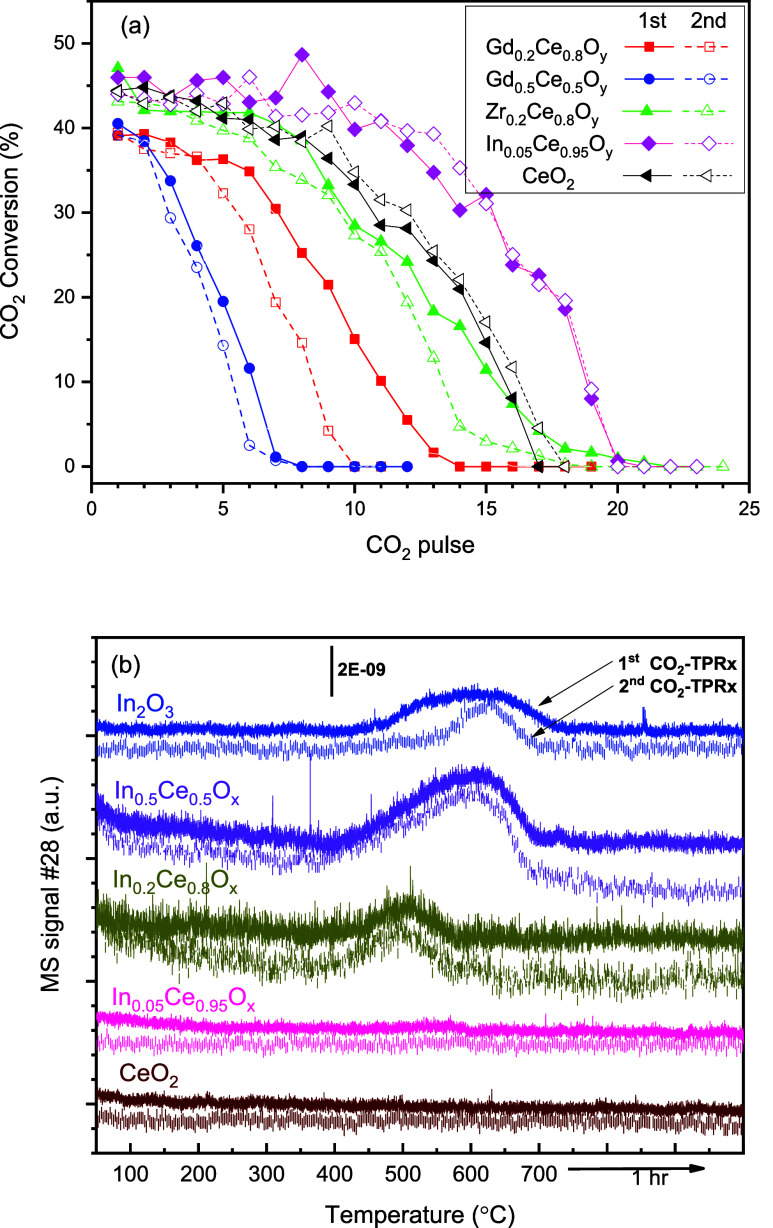
CO_2_ deoxygenation results of selected samples in 2-cycle tests using (a) an isothermal pulse input of 5% CO_2_ at 700 °C after the sample was activated by TPR up to 900 °C and (b) TPRx from 50 to 700 °C under a 5% CO_2_ flow after the sample was activated by TPR up to 700 °C.

A quick glance of the presented CO_2_ deoxygenation capacity listed in Table 1 indicates that In was an effective dopant to enhance the CO_2_ deoxygenation, while the other three dopants, namely, Zr, Gd, and Sm, were relatively ineffective. All of the CO_2_ deoxygenation tests listed in Table 1 had CO as the only observed gas-phase product, and none of the spent samples exhibited an observable coke deposit in the TGA test. The quantitatively calculated CO yield was typically around 0.95. The TPRx test was less sensitive in detecting the deoxygenation capacity than the isothermal pulse input test, e.g., as that observed with undoped CeO_2_ and In_0.05_Ce_0.95_O_*y*_. This could be due to the more noisy background in the TPRx test, as indicated in Figure 2. However, the TPRx test could provide a quick scan of the reactivity within the test temperature range. Figure 2 shows the results of selected samples by using the two types of CO_2_ deoxygenation tests. The trend observed in the isothermal pulse input test results (Figure 2a) has been discussed in our previous report.^7^ Briefly, the initial CO_2_ pulses exhibited a near-constant per-pulse conversion, which can be explained as owing to a CO_2_ deoxygenation over surface vacancies coupled with a quick exchange between bulk and surface vacancies and the surface reaction is considered the rate-determining step. This can lead to a near-constant surface status and the observed nearly constant per-pulse conversion. However, the per-pulse CO_2_ conversion would eventually start to gradually decay until zero activity, attributable to the inward migration of stripped oxygen eventually leading to near saturation of available bulk vacancies and a consequent suppression of the reactivity. Figure 2a shows the isothermal pulse input test results of selected samples. Both Zr- and Gd-doped CeO_2_, at 20 or 50%(mol) doping concentrations led to an inferior reactivity at 700 °C than undoped CeO_2_. In_0.05_Ce_0.95_O_*y*_ readily showed a higher reactivity than undoped CeO_2_ and the influence of the In doping concentration was further examined with a TPRx test, as shown in Figure 2b. Gd_0.5_Ce_0.5_O_*x*_ had the lowest deoxygenation capacity and the quickest decay, as shown in Figure 2a, suggesting that its surface activity was more significantly influenced by the subsurface vacancies. The results of isothermal pulse input tests listed in Table 1 indicate that the oxygen vacancies generated after 900 °C TPR can catalyze CO_2_ deoxygenation at as low as 500 °C but the deoxygenation capacity would be decreased by the decrease in the reaction temperature, e.g., that of CeO_2_, Zr_0.2_Ce_0.8_O_*y*_, and In_0.05_Ce_0.95_O_*y*_, as listed in Table 1. With the TPRx tests, only In_*x*_Ce_1–*x*_O_*y*_ (*x* = 0.2 and 0.5) samples presented an observable CO_2_ deoxygenation (Table 1 and Figure 2b). The two active In_*x*_Ce_1–*x*_O_*y*_ showed an onset deoxygenation temperature at around 400 °C. In_2_O_3_ by itself showed activity, as shown in Figure 2b, but it exhibited a higher deoxygenation onset temperature, a lower per gram deoxygenation capacity, and a poorer stability comparing to active In_*x*_Ce_1–*x*_O_*y*_ under the same test conditions. The fact that In_0.5_Ce_0.5_O_*y*_ had a significantly higher deoxygenation capacity than In_0.2_Ce_0.8_O_*y*_ implies the important role of In. The XRD analyses reported later showed the exsolution of In from the fluorite framework after TPR. This suggests a synergy between exsoluted In and the fluorite substrate in the two active In_*x*_Ce_1–*x*_O_*y*_ samples. The deoxygenation capacity of active In_*x*_Ce_1–*x*_O_*y*_ was found not significantly influenced by decreasing the TPR treatment temperature from 900 to 700 °C, indicating that the surface-active sites could be readily generated after TPR to 700 °C.

Three indexes are included in Table 1 to discuss the TPR and deoxygenation stability/reversibility of the examined M_*x*_Ce_1–*x*_O_*y*_ samples during 2 cycles of TPR-CO_2_-deoxygenation tests. The index O_2_^second^/O_2_^first^ indicates the ratio of the deoxygenation capacity of the second cycle to that of the first cycle and a ratio of 1 indicates an ideally reproduced second cycle deoxygenation capacity as in the first cycle. Among the active M_*x*_Ce_1–*x*_O_*y*_ samples, Gd- and Zr-doped samples exhibited relatively low O_2_^second^/O_2_^first^, while the undoped and In-doped samples could have a number near 1. All Gd-doped and Zr-doped CeO_2_ samples listed in Table 1 had this index of 0.8 or less, indicating a decrease in the deoxygenation capacity. This implies that Gd_*x*_Ce_1–*x*_O_*y*_ and Zr_*x*_Ce_1–*x*_O_*y*_ were not stable, though active, in such a sequential TPR-deoxygenation test. The second index H_2_^second^ /H_2_^first^ listed in Table 1 represents the ratio of TPR hydrogen consumption (Table S2) in the second cycle to that in the first cycle. We discussed in our previous work about the presence of irreversible and reversible oxygen vacancies in CeO_2_,^7^ which can be revealed using this H_2_^second^/H_2_^first^ index. Most samples, except In_0.5_Ce_0.5_O_*y*_, exhibited a number less than 1, indicating a lower H_2_ consumption in the second TPR than that in the first TPR wherein the presence of irreversible oxygen vacancies in the first TPR is suggested. The third index H_2_^second^/O_2_^first^ listed in Table 1 indicates the fraction of the stripped oxygen (i.e., deoxygenation capacity) in the first cycle that can be removed by hydrogen again in the second TPR, and a number of 2 would indicate that all of the stripped oxygen from the first CO_2_ deoxygenation can be completely removed by the second TPR. From Table 1, this index appears to be dependent on the TPR treatment temperature as that observed with undoped CeO_2_ and Zr_0.2_Ce_0.8_O_*y*_. Most tested samples listed in Table 1 had an H_2_^second^/O_2_^first^ near 2, indicating that the stripped oxygen from CO_2_ can be mostly removed again by H_2_ during TPR. This suggests the reversible formation of oxygen vacancies. In summary, the two active In_*x*_Ce_1–*x*_O_*y*_ samples appeared superior to the other tested samples in having good reactivity, nearly reversible vacancy formation, and a reversible deoxygenation capacity.

### 3.2 Characterization of In_*x*_Ce_1–*x*_O_*y*_

**Figure 3:**
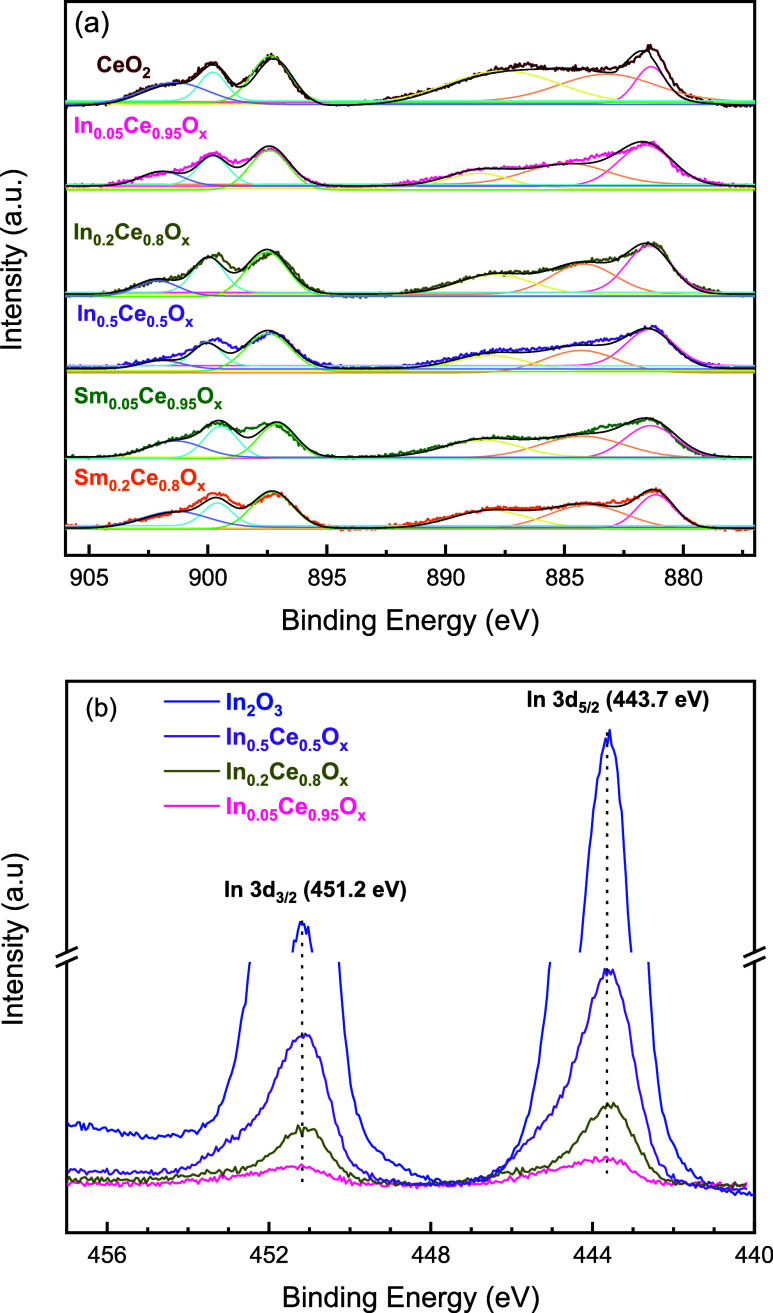
Ex situ XPS spectra of selected as-prepared samples: (a) Ce 3d, showing the fitting results of the combination of Ce^3+^ and Ce^4+^ (fitting results are shown in Table S2), and (b) In 3d.

From the above discussions, it is noted that In_*x*_Ce_1–*x*_O_*y*_ samples, especially the one with *x* = 0.5, exhibited a relatively high and reversible deoxygenation capacity in the sequential TPR-CO_2_ deoxygenation test with an onset temperature of CO_2_ deoxygenation as low as 400 °C. The as-prepared In_*x*_Ce_1–*x*_O_*y*_ samples were analyzed by XPS in comparison to the relatively inactive undoped CeO_2_ and Sm_*x*_Ce_1–*x*_O_*y*_ samples. The spectra of Ce 3d and In 3d of the as-prepared samples are shown in Figure 3. The Ce 3d spectra (Figure 3a) were fitted as the combination of Ce^3+^ and Ce^4+^ and the fitting results are shown in Table S3. The as-prepared In_*x*_Ce_1–*x*_O_*y*_ samples did not reveal an increase in the Ce^3+^/Ce^4+^ ratio, neither did Sm_*x*_Ce_1–*x*_O_*y*_ samples, comparing to undoped CeO_2_ (Table S3). The Ce M_4,5_ edge XANES (X-ray absorption near-edge spectroscopy) also shows an insignificant difference among the as-prepared In_*x*_Ce_1–*x*_O_*y*_, Sm_*x*_Ce_1–*x*_O_*y*_, and undoped CeO_2_ samples (Figure S1). This implies that the Ce^3+^/Ce^4+^ ratio of the as-prepared samples is not a governing factor of the CO_2_ deoxygenation activity, as CeO_2_ and Sm_*x*_Ce_1–*x*_O_*y*_ samples were relatively inactive compared to In_*x*_Ce_1–*x*_O_*y*_ (Table 1). The calculated In/Ce ratio from XPS spectra of as-prepared In_*x*_Ce_1–*x*_O_*y*_ samples was found to be consistent with the experimental bulk composition. This, together with the absence of the impurity phase in XRD (Figure 1), suggests that the as-prepared In_*x*_Ce_1–*x*_O_*y*_ samples were homogeneous solid mixtures with In doping into the fluorite framework. Figure 3b shows that the XPS In 3d spectra of as-prepared In_*x*_Ce_1–*x*_O_*y*_ samples had a negligible peak shift in comparison to In_2_O_3_, which confirmed In^3+^ doping into the CeO_2_ lattice in the as-prepared In_*x*_Ce_1–*x*_O_*y*_ samples.

**Figure 4:**
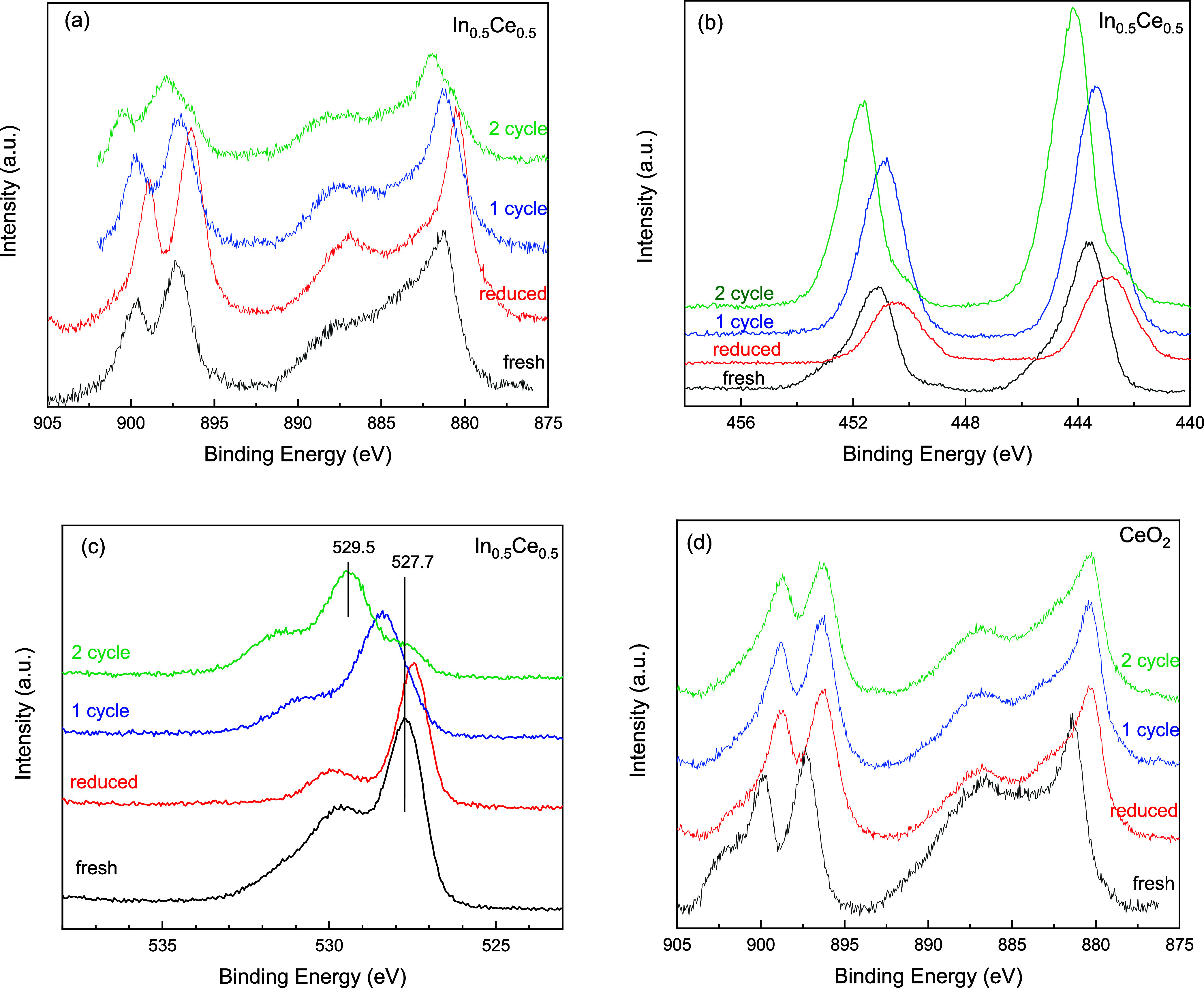
Ex situ XPS analysis of In_0.5_Ce_0.5_O_*y*_ and CeO_2_ samples at different stages during 2 cycles of the TPR deoxygenation sequential test. (a) Ce 3d, (b) In 3d, (c) O 1s of In_0.5_Ce_0.5_O_*y*_, and (d) Ce 3d of CeO_2_. Fresh: as-prepared, reduced: after 1st TPR, 1 cycle: after 1st TPR and 1st TPRx deoxygenation, and 2 cycle: after 2nd TPR and 2nd TPRx deoxygenation. All XPS data were analyzed after sample cooling to room temperature and then exposure to air.

Figure 4 shows the comparison of ex situ XPS Ce 3d, In 3d, and O 1s spectra of In_0.5_Ce_0.5_O_*y*_ when as-prepared, reduced (after the first 700 °C TPR), after the first cycle (TPR and CO_2_ deoxygenation) test, and after the second cycle test, with undoped CeO_2_ as the reference. After 700 °C TPR, reduced In_0.5_Ce_0.5_O_*y*_ showed shifts of both Ce 3d (Figure 4a) and In 3d (Figure 4b) toward a lower binding energy, indicating an increase in Ce^3+^ and reduced In. After the CO_2_ deoxygenation test, both Ce 3d and In 3d would shift in reverse toward a higher binding energy, indicating the oxidation of both. The sample after the first cycle test and that after the second cycle test similarly revealed the more oxidized state in Ce 3d and In 3d than the reduced one, though the shifts after the first cycle and that after the second cycle were not exactly the same. The results indicate that the redox of both Ce and In occurred during the sequential TPR-CO_2_ deoxygenation test with In_0.5_Ce_0.5_O_*y*_. The observed oxidation of reduced In by CO_2_ is similar with the reported presence of surface oxygen when polycrystalline In^0^ was exposed to CO_2_.^12^Figure 4c shows the XPS O 1s of In_0.5_Ce_0.5_O_*x*_, while that of CeO_2_ is shown in Figure S2a. As-prepared In_0.5_Ce_0.5_O_*x*_ revealed a main O 1s peak at 527.7 eV, at a somewhat higher binding energy than the main O 1s peak observed of as-prepared CeO_2_ (at 527.2 eV, Figure S2a). After reduction, the O 1s peak of In_0.5_Ce_0.5_O_*x*_ shifted to a lower binding energy near that observed with undoped CeO_2_. This suggests that the In doping to the CeO_2_ lattice in as-prepared In_0.5_Ce_0.5_O_*x*_ influenced the lattice O binding energy, while In exsolution caused the shift in the CeO_2_ lattice O binding energy closer to that of undoped CeO_2_. After the CO_2_ deoxygenation test, the O 1s of In_0.5_Ce_0.5_O_*x*_ appeared as a combination of multiple peaks and the major peak after the 2-cycle test appeared at 529.5 eV, which is close to the reported O 1s position of the lattice oxygen of In_2_O_3_.^13,14^ The peaks observed at a higher O 1s binding energy than the lattice oxygen can be due to O_2_ adsorption on vacancies and formation of surface groups such as hydroxyl, carbonates, etc.^13−15^ In comparison, the O 1s of CeO_2_ did not show a significant peak shift through the 2-cycle test in this study, as shown in Figure S2a. Figure 4d shows that CeO_2_ also had a shift in Ce 3d after reduction toward a lower binding energy, but no obvious reverse shift was noted after the first and second cycle tests. This indicates that Ce^3+^ induced by the TPR of CeO_2_ could not be oxidized by CO_2_ under the conditions of our tests, consistent with its low reactivity in the deoxygenation test. The relatively inactive Sm_0.5_Ce_0.5_O_*y*_ also did not reveal a Ce^4+^–Ce^3+^ redox reversibility (Figure S2). The XPS results clearly indicate that In doping leads to an enhanced redox reversibility of the CeO_2_-like fluorite framework and that exsoluted In was also involved in the redox reversibility.

**Figure 5:**
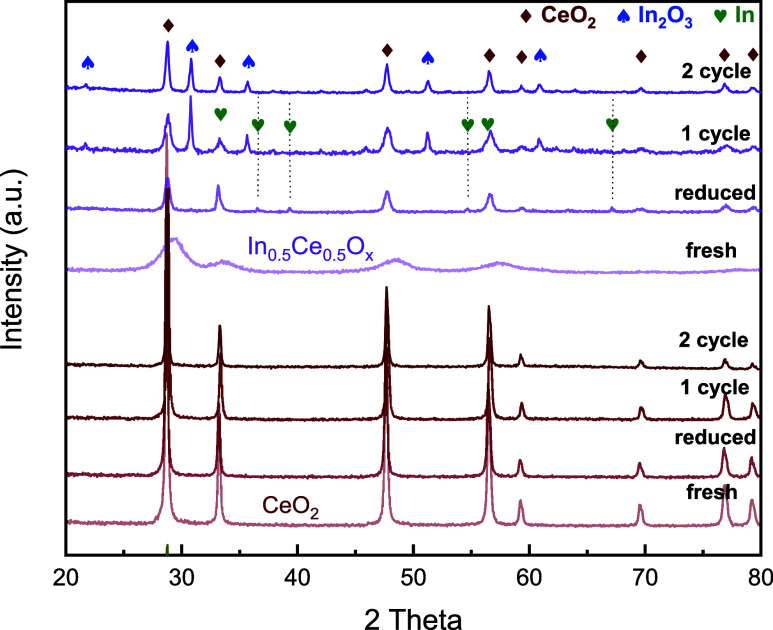
XRD analysis of CeO_2_, In_0.2_Ce_0.8_O_*y*_, and In_0.5_Ce_0.5_O_*y*_ samples at different stages during 2 cycles of the TPR deoxygenation sequential test. Fresh: as-prepared, reduced: after 1st TPR, 1 cycle: after 1st TPR (to 700 °C) and 1st TPRx deoxygenation, and 2 cycle: after 2nd TPR and 2nd TPRx deoxygenation. All XRD data were analyzed after sample cooling to room temperature and then exposure to air.

The structure of In_0.5_Ce_0.5_O_*y*_ as-prepared, reduced (after the first TPR), after the first cycle test, and after the second cycle test was examined by ex situ XRD, as shown in Figure 5. The reduced In_0.5_Ce_0.5_O_*y*_ revealed an In^0^ phase even though the samples were exposed to air for XRD measurement. This indicates that doped In became reduced and leached from the fluorite framework during TPR, likely following a so-called exsolution process. We did not observe such an exsolution phenomenon in XRD when with the other dopants after TPR in this study. After CO_2_ deoxygenation, the In_2_O_3_ phase could be found, while In^0^ disappeared, which corroborates the redox of In observed in XPS. The XRD results indicate that the exsoluted In^0^ phase participated in CO_2_ deoxygenation and became oxidized by the oxygen stripped from CO_2_. The In_2_O_3_ phase was continuously observed after the second cycle of the TPR deoxygenation test though its diffraction peak positions were somewhat shifted compared to that after the first cycle, which seems consistent with the somewhat different oxidation state of In observed in XPS (Figure 4). The different peak shift may be related to changes in bonding environments, such as that influenced by nanocrystalline in XRD-amorphous In_2_O_3_ films, as reported by Buchholz et al.^16^ The In_0.2_Ce_0.8_O_*y*_ and pure In_2_O_3_ samples also revealed the redox of the In phase during the sequential TPR and CO_2_ deoxygenation test, as indicated by the XRD analyses, as shown in Figure S3. The In^0^ phase observed in the reduced In_2_O_3_ sample disappeared after the CO_2_ deoxygenation (Figure S3b). The results indicate that exsoluted In^0^ participated in the CO_2_ deoxygenation reaction.

A closer look at the XRD patterns shows that the CeO_2_(111) peak position varied along the TPR reduction and CO_2_ deoxygenation tests. In_0.5_Ce_0.5_O_*y*_ exhibited a relatively small variation in its CeO_2_(111) peak position, compared to that of CeO_2_ and In_0.2_Ce_0.8_O_*y*_ (Figure S3a). This suggests that In_0.5_Ce_0.5_O_*y*_ could maintain a relatively stable fluorite crystal lattice during the 2-cycle sequential TPR and CO_2_ deoxygenation test, implying a relatively more reversible lattice change during the redox of the fluorite phase. The In_2_O_3_ phase of In_*x*_Ce_1–*x*_O_*y*_ after CO_2_ deoxygenation had somewhat shifted peak positions in the 1-cycle and 2-cycle samples, but the extent of shift in In_0.5_Ce_0.5_O_*y*_ was smaller than that of In_0.2_Ce_0.8_O_*y*_ (Figure S3a). In_0.5_Ce_0.5_O_*y*_ appears to have a somewhat better structure stability during the redox induced by sequential TPR-CO_2_ deoxygenation.

**Figure 6:**
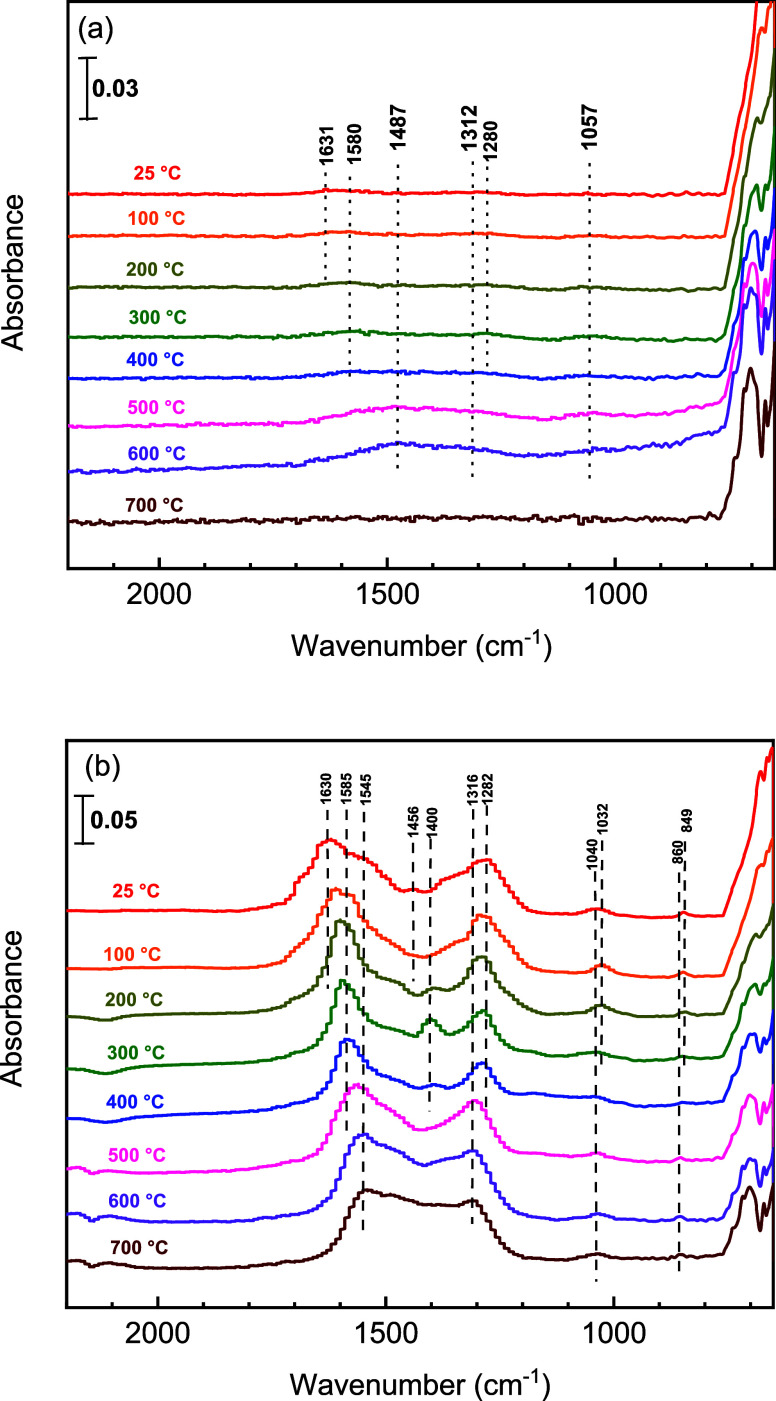
In situ DRIFTS analysis during CO_2_-TPRx over (a) reduced In_0.5_Ce_0.5_O_*y*_ and (b) reduced CeO_2_. Both samples were in line with reduced samples by TPR up to 700 °C. The background spectra were recorded over reduced samples under Ar at the specified temperature of the spectrum.

In situ DRIFTS was applied to examine the evolution of surface species during stepwise CO_2_-TPRx over In_0.5_Ce_0.5_O_*y*_ and CeO_2_ samples after in-line reduction by TPR to 700 °C. Figure 6 shows that In_0.5_Ce_0.5_O_*y*_ exhibited weak absorbance intensities compared to CeO_2_. In the 400–600 °C temperature range when we observed deoxygenation activity over In_0.5_Ce_0.5_O_*y*_ (Figure 2b), monodentate carbonate (broad bands at 1057, 1315, and 1487 cm^–1^) could be barely observed, while no CO-related band was found. The weak absorbance intensities in the DRIFTS spectra imply a low surface CO_2_ coverage over reduced In_0.5_Ce_0.5_O_*y*_, and the surface sites could catalyze the CO_2_ deoxygenation when thermally activated. This seems consistent with the proposed rate-determining surface deoxygenation reaction coupled with a fast inward diffusion of stripped oxygen.^7^ In_2_O_3_ after TPR also exhibited weak absorbance intensities during CO_2_-TPRx (Figure S4a) though surface carbonate species were observable at room temperature, which is consistent with the report by Gericke et al.^17^ This indicates a weak CO_2_ adsorption over the reduced In surface. In comparison, the surface carbonate adspecies from CO_2_ were more observable at room temperature over reduced CeO_2_ (Figure 6b) and Sm_0.5_Ce_0.5_O_*y*_ (Figure S4b), whose band intensities decreased and shifted at high temperatures, different from that observed over In_0.5_Ce_0.5_O_*y*_. It is not known if the strong adsorption over these two samples leads to low surface reactivity or vice versa. The band assignments of the DRIFTS spectra are listed in Table S4. In general, CeO_2_, Sm_0.5_Ce_0.5_O_*y*_, and In_2_O_3_ samples all followed a similar pattern of surface species evolution, i.e., the presence of bicarbonate, bidentate carbonate, and monodentate carbonate species at room temperature, followed by a sequential decrease in bicarbonate and bidentate carbonate with increasing temperatures. Monodentate carbonate became obvious at high temperatures. Overall speaking, In_0.5_Ce_0.5_O_*y*_ revealed a somewhat different pattern of surface species evolution compared to the other samples examined in this study, showing very weak absorbance band intensities at room temperature.

In_2_O_3_-based catalysts are considered promising for CO_2_ hydrogenation to methanol^18^ and oxygen vacancies are frequently considered to play an important role. A density functional theory (DFT) study suggests that CO_2_ adsorption occurs over vacancies on In_2_O_3_(110).^19^ On the other hand, CO_2_ adsorption was reported over In_2_O_3_(111), oxidized, reduced, and hydroxylated, based on XPS and DFT, wherein vacancies were considered not a key factor.^17^ Furthermore, very low CO_2_ adsorption was found over In_2_O_3_(111) at above 298 K even when with oxygen vacancies.^17^ In_2_O_3_/ZrO_2_ reported active for CO_2_ hydrogenation to methanol showed absorbance bands in the range 900–1100 cm^–1^ in DRIFTS, which were attributed to CO_2_ over vacancies.^8^ Our DRIFTS results did not reveal a related CO_2_ adsorption band in this range. No previous infrared study reported the carbonate-related bands over In_2_O_3_ in the range 1100–1600 cm^–1^, to the best of our knowledge, as in this study over TPR-reduced In_0.5_Ce_0.5_O_*y*_. These results suggest that the adsorption of CO_2_ over In_2_O_3_ may be structure-dependent and that our TPR-reduced In_0.5_Ce_0.5_O_*y*_ sample did not favor CO_2_ adsorption. The other possible reason is that the exsoluted indium phase contained mainly In^0^ on the surface after TPR and In^0^ does not favor CO_2_ adsorption. Gericke et al.^17^ also mentioned that In adatoms on reduced In_2_O_3_(111) seemed not to influence CO_2_ adsorption. Notably, the CeO_2_-like phase after In exsolution from In_0.5_Ce_0.5_O_*y*_ by TPR also showed weak CO_2_ adsorption, not like the observed obvious carbonate-related surface species over TPR-reduced CeO_2_ (Figure 6b) and Sm_0.5_Ce_0.5_O_*y*_ (Figure S4b). This suggests a different CeO_2_-like surface after In exsolution or that exsoluted In^0^ would block the adsorption sites over the CeO_2_ surface.

**Figure 7:**
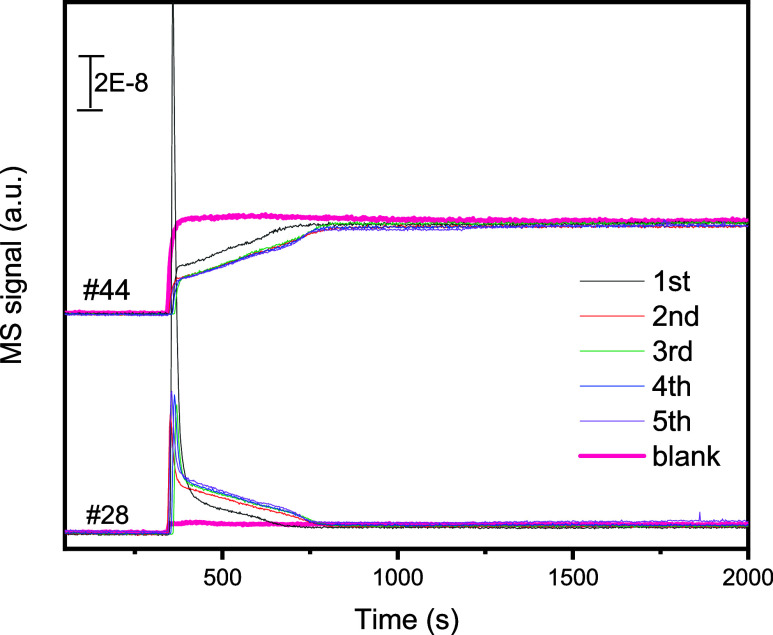
Mass spectrometry signals during 5 cycles of isothermal CO_2_ deoxygenation in 5 consecutive cycles of the sequential H_2_-reduction-CO_2_-deoxygenation test over In_0.5_Ce_0.5_O_*x*_ at 700 °C. The “blank” indicates the results in the absence of In_0.5_Ce_0.5_O_*x*_ (i.e., empty tube).

An isothermal sequential H_2_-reduction-CO_2_-deoxygenation was performed with In_0.5_Ce_0.5_O_*x*_ at 700 °C to evaluate its stability. Figure 7 shows the MS signals from the test. A reasonably good stability was found in 5 consecutive cycles. The quantitatively calculated H_2_ consumption, CO_2_ consumption, and CO formation are shown in Table S5. The results of this study indicate that In_0.5_Ce_0.5_O_*x*_ showed a good potential for CO_2_ thermal deoxygenation to CO, with a deoxygenation activity and capacity contributed from both the exsoluted In phase and the accompanied CeO_2_-like fluorite phase. The deoxygenation occurred with an onset temperature around 400 °C, which can be compatible with the midtemperature oxygen-conducting membrane reactor for facilitated transport of stripped oxygen from CO_2_. Furthermore, In_0.5_Ce_0.5_O_*x*_ revealed interesting redox behaviors during sequential TPR and CO_2_ deoxygenation, involving both exsoluted In^0^ and the accompanied CeO_2_-like fluorite, as evidenced from both the XPS-analyzed surface property and the XRD-analyzed crystal structure. We previously proposed that the CO_2_ deoxygenation involves a surface reaction coupled with an inward diffusion of stripped oxygen, with the surface reaction considered the rate-determining step, for the CO_2_ deoxygenation over reduced CeO_2_ at 700 °C.^7^ In-doped CeO_2_ has been reported to have an enhanced oxygen-ion conductivity compared to CeO_2_.^20^ This enhanced oxygen-ion conductivity might partially contribute to the observed CO_2_ deoxygenation activity. However, Sm doping to CeO_2_ is well known to induce an enhanced oxygen-ion conductivity of CeO_2_ but Sm_0.5_Ce_0.5_O_*y*_ did not show significant CO_2_ deoxygenation activity in our test. This indicates that the reactivity of surface sites may be more important.

We quantitatively analyzed the H_2_ consumption of In_0.2_Ce_0.8_O_*y*_ and In_0.5_Ce_0.5_O_*y*_ during the two TPR steps in our sequential test cycles (Table S6). The results are compared to that theoretically needed to reduce In^3+^ to In^0^, from which approximately 75% of In in In_0.5_Ce_0.5_O_*y*_ was estimated to be exsoluted and reduced during TPR when assuming that all of the consumed H_2_ was used for In^3+^ reduction. This suggests that the remaining CeO_2_-like phase after In exsolution still contained a fraction of the In dopant, but the exact structure could not be unequivocally resolved. At this moment, we could not identify the possible surface structure of active TPR-reduced In_0.5_Ce_0.5_O_*y*_. However, XPS analyses indicated that the redox of both In and Ce occurred over In_0.5_Ce_0.5_O_*y*_ during our TPR-CO_2_-TPRx sequential test. The observed deoxygenation capacity over In-doped CeO_2_ was contributed by both the oxygen vacancies of the CeO_2_-like phase and the redox of the exsoluted In phase. The redox measurement typically applied for estimating the OSC (oxygen storage capacity) could not distinguish these two contributions. Considering that undoped CeO_2_ and that doped with Gd, Sm, and Zr samples were inactive during CO_2_-TPRx in this study, a possible scenario is that reduced In played as the active surface and its oxidation coupling with the migration of O_stripped_ to the CeO_2_-like phase contributed to the observed deoxygenation capacity. On the other hand, the highly dispersed metal, down to the single atom, over oxide substrates is reported active and can produce a selective product during CO_2_ hydrogenation.^21−24^ We cannot exclude such a possibility of In clusters over the fluorite substrate contributing to the observed CO_2_ deoxygenation activity. Though we do not have direct evidence to support the claimed scenario, the results point to a synergistic effect between In and the CeO_2_-like surface.

## 4 Conclusions

In this study, M-doped CeO_2_ (M = Zr, Gd, Sm, In) is prepared and confirmed as homogeneous solid mixtures. After H_2_-TPR, only In-doped CeO_2_ exhibits significant CO_2_ deoxygenation capability, with an onset temperature of deoxygenation at 400 °C during CO_2_-TPRx. In_0.5_Ce_0.5_O_*y*_ shows a higher deoxygenation capacity than those of In_0.2_Ce_0.8_O_*y*_ and In_0.05_Ce_0.95_O_*y*_. XRD analyses indicate that In is leached from the fluorite framework, forming In^0^ during H_2_-TPR, and In^0^ can be oxidized during CO_2_ deoxygenation. In comparison, In_2_O_3_ shows a similar redox in the H_2_-TPR and CO_2_-TPRx sequential test, but it has a lower CO_2_ deoxygenation capacity and a higher onset temperature for CO_2_ deoxygenation than In_0.5_Ce_0.5_O_*y*_. Both XRD and XPS indicate that In_0.5_Ce_0.5_O_*y*_ has a reversibly occurred redox of both In and fluorite phases during the H_2_-TPR and CO_2_-TPRx sequential test. DRIFTS reveals a low CO_2_ uptake over In_0.5_Ce_0.5_O_*y*_, indicating that the enhanced deoxygenation capability can be attributed mainly to enhanced surface reactivity. The results of this study suggest that a thermolytic CO_2_ deoxygenation to CO can be possible with the potential of integration with the midtemperature oxygen-conducting oxide membrane.

## Supporting Information Available

Morphological properties of as-prepared samples (Tables S1 and S3 and Figure S1); evolution of sample properties along the test protocol (Figures S2 and S3); in situ DRIFTS during CO_2_-TPRx (Figure S4 and Table S4); and the calculated consumption of H_2_ and CO_2_ during the test protocol (Tables S2, S5, and S6) (PDF)
