# Supplementary material for: Midtemperature CO2 Deoxygenation to CO over Oxygen Vacancies of Doped CeO2
Source: ACS Appl Mater Interfaces. 2025 May 1;17(19):28163–72. doi: 10.1021/acsami.4c17644 (PMC12086774; doi:10.1021/acsami.4c17644)
Supplement: Supplementary file 1 — am4c17644_si_001.pdf [file am4c17644_si_001.pdf]

Supporting Information

Mid-temperature CO_2 deoxygenation to CO over oxygen vacancies of doped CeO_2

Nan-Chian Chiang, Tz-Jie Ju, Yi-Cheng Wang, Tzu-Peng Lin, Jia-Han Guo, and Shawn D. Lin *

Department of Chemical Engineering, National Taiwan University of Science and Technology, Taipei 10617, Taiwan

[illegible] Corresponding author e-mail address: sdlin@mail.ntust.edu.tw

Table S1. The morphological properties of as prepared $M_xCe_{1-x}O_y$ samples.

Sample	Crystallite size [1] (nm)	BET area (m^2/g)	Pore size (nm)
CeO_2	33.6	6	11.8
$Gd_{0.2}Ce_{0.8}O_y$	9.0	62	5.3
$Gd_{0.5}Ce_{0.5}O_y$	7.1	43	6.0
$Zr_{0.2}Ce_{0.8}O_y$	7.5	89	5.0
$Zr_{0.5}Ce_{0.5}O_y$	5.2	3	5.5
$Sm_{0.05}Ce_{0.95}O_y$	16.5	32	9.1
$Sm_{0.2}Ce_{0.8}O_y$	16.9	28	9.7
$Sm_{0.5}Ce_{0.5}O_y$	16.9	28	10.1
$In_{0.05}Ce_{0.95}O_y$	6.1	99	5.0
$In_{0.2}Ce_{0.8}O_y$	5.3	70	2.7
$In_{0.5}Ce_{0.5}O_y$	3.7	96	3.5
In_2O_3	13.2	68	5.3

[1] Calculated by Scherrer equation using Warren peak width correction, based on the diffraction peak of fluorite (111). The crystallite size of In_2O_3 was evaluated based on In_2O_3 (222) peak.

Table S2. Calculated hydrogen consumptions during TPR of the sequential TPR-CO_2 deoxygenation test over $M_xCe_{1-x}O_y$ samples, mostly of two consecutive cycles.

Sample	T_{TPR} [1] (°C)	T_{CO2} [2] (°C)	$H_{2, consumed}$ (μmol H_2/g) 1st cycle	2nd cycle	H_2^{2nd} / H_2^{1st}	H_2^{2nd} / O_2^{1st}
CeO_2	900	700	105	0.63	0.63	2.0
	900	500	94	0.57	0.57	2.0
	900	50-700	87	0.89	0.89	--
	700	700	48	0.31	0.31	--
	700	50-700	60	0.98	0.98	--
$Gd_{0.2}Ce_{0.8}O_y$	900	700	42	0.90	0.90	1.9
	700	50-700	14	--	--	--
$Gd_{0.5}Ce_{0.5}O_y$	900	700	32	0.72	0.72	2.1
$Zr_{0.2}Ce_{0.8}O_y$	900	700	102	0.66	0.66	2.1
	900	500	84	0.64	0.64	--
	700	700	43	0.56	0.56	--
	700	50-700	79	--	--	--
$Zr_{0.5}Ce_{0.5}O_y$	900	700	72	0.86	0.86	3.1
	700	50-700	48	--	--	--
$Sm_{0.05}Ce_{0.95}O_y$	900	50-700	498	--	--	--
$Sm_{0.2}Ce_{0.8}O_y$	900	50-700	477	--	--	--

$Sm_{0.5}Ce_{0.5}O_y$	900	50-700	594	--	--	--
$In_{0.05}Ce_{0.95}O_y$	900	700	121	0.70	0.70	2.0
	900	500	106	0.75	0.75	2.0
	900	50-700	529	0.57	0.57	--
	700	700	66	0.48	0.48	2.0
	700	50-700	648	0.48	0.48	--
$In_{0.2}Ce_{0.8}O_y$	900	50-700	1527	0.90	0.90	1.8
	700	50-700	1562	0.93	0.93	1.9
$In_{0.5}Ce_{0.5}O_y$	900	50-700	3501	0.98	0.98	2.0
	700	50-700	3533	0.98	0.98	2.0
In_2O_3	700	50-700	2608	0.80	0.80	1.9

[1] TPR treatment with a upper limit temperature of either 700 or 900 ^{o}C, and the calculated corresponding H_2 consumption.

[2] CO_2 deoxygenation reaction carried out either isothermally with pulse input of 5% CO_2 or by a TPRx from 50 to 700 ^{o}C under a 5% CO_2 flow, and the calculated corresponding stripped oxygen.

Table S3. Calculated Ce^{3+} fraction from the fitting of XPS Ce 3d spectra of as-prepared samples.

Sample	Ce^{3+} (%)
CeO_2	34.7
$In_{0.05}Ce_{0.95}O_x$	32.8
$In_{0.2}Ce_{0.8}O_x$	27.0
$In_{0.5}Ce_{0.5}O_x$	25.3

$Sm_{0.05}Ce_{0.95}O_x$	33.2
$Sm_{0.2}Ce_{0.8}O_x$	35.6
$Sm_{0.5}Ce_{0.5}O_x$	20.7

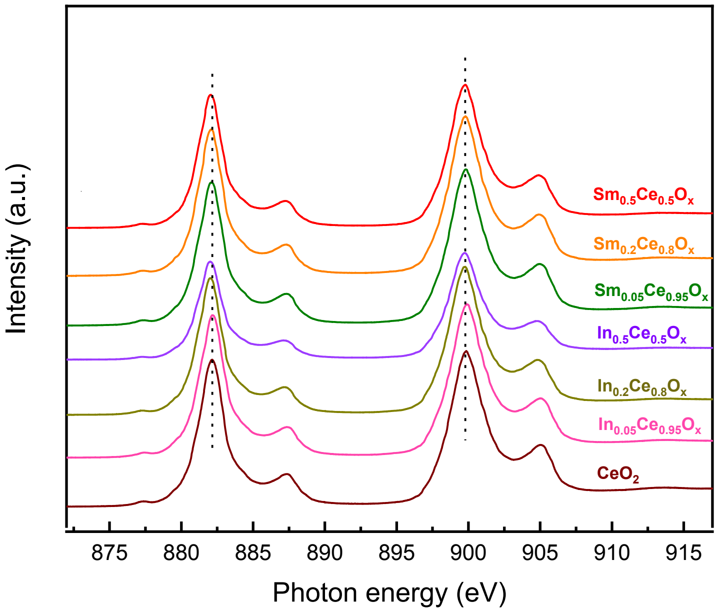

Figure S1. Ce $M_{4,5}$ edge XANES (X-ray absorption near edge spectroscopy) of as-prepared $In_xCe_{1-x}O_y$, $Sm_xCe_{1-x}O_y$, and undoped CeO_2 samples. The analyses were performed using Beamline 20A of the National Synchrotron Radiation Research Center (NSRRC), Taiwan.

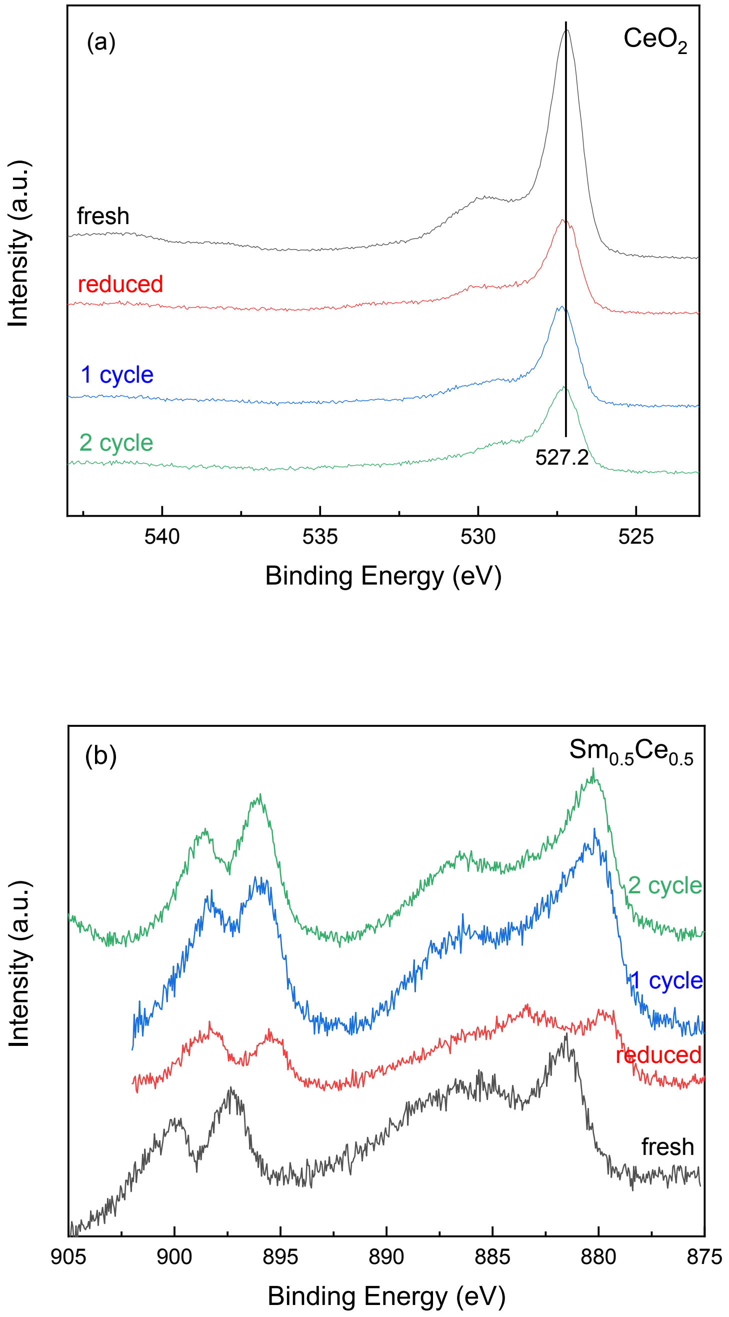
(a)
CeO2
fresh
reduced
1 cycle
2 cycle
527.2
Intensity (a.u.)
540
535
530
525
Binding Energy (eV)
(b)
Sm0.5Ce0.5
2 cycle
1 cycle
reduced
fresh
Intensity (a.u.)
905
900
895
890
885
880
875
Binding Energy (eV)

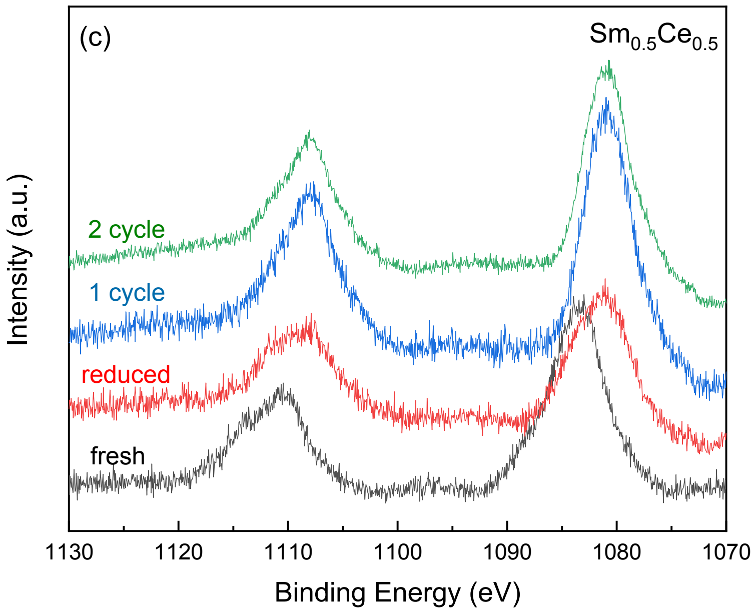

Figure S2. Ex situ XPS analysis of undoped CeO_2 and $Sm_{0.5}Ce_{0.5}O_y$ sample at different stage during 2 cycles of TPR-deoxygenation sequential test, (a) O 1s of CeO_2, (b) Ce 3d, and (c) Sm 3d of $Sm_{0.5}Ce_{0.5}O_y$. fresh: as-prepared, reduced: after 1st TPR, 1 cycle: after 1st TPR and 1st TPRx deoxygenation, and 2 cycle: after 2nd TPR and 2nd TPRx deoxygenation. All XPS were analyzed after sample cooling to room temperature and then exposure to air.

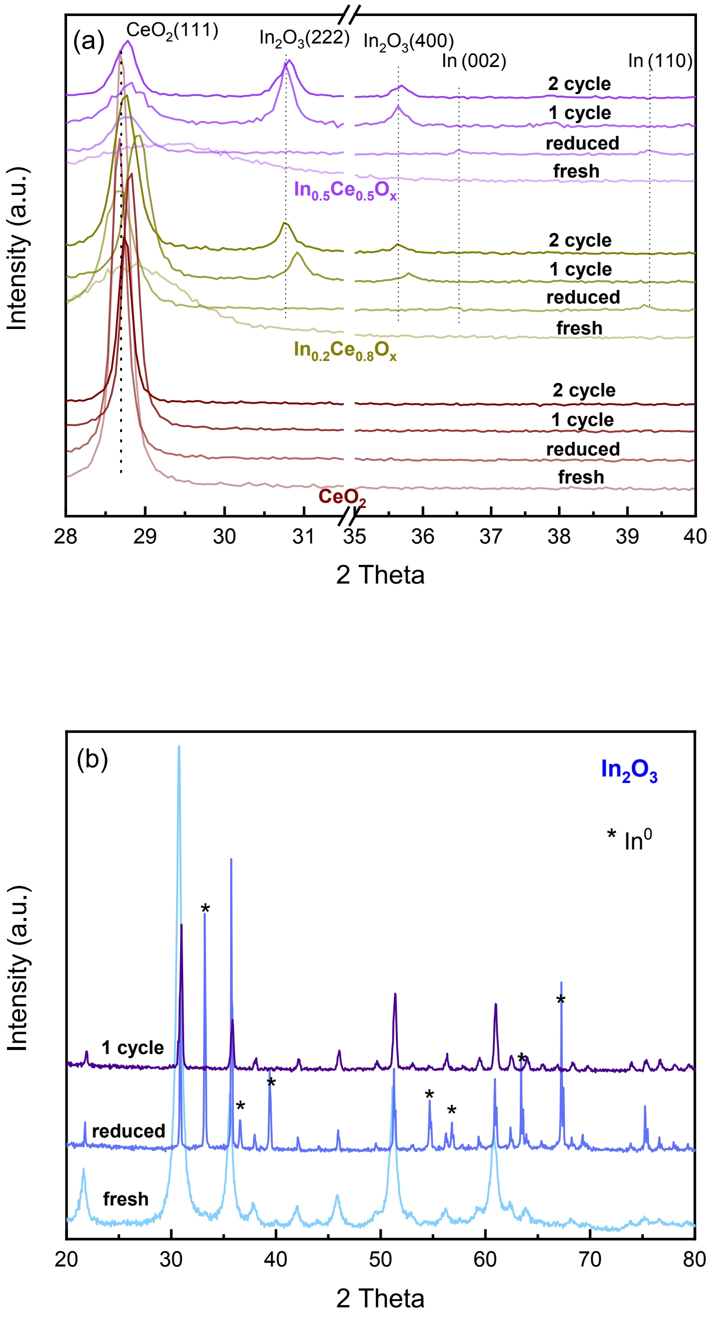

Figure S3. XRD analysis of (a) CeO_2, $In_{0.2}Ce_{0.8}O_y$, and $In_{0.5}Ce_{0.5}O_y$ samples, and (b) In_2O_3 sample at different stage during TPR-deoxygenation sequential test. fresh: as-prepared, reduced: after 1st TPR (to 700 ^{o}C), and 1 cycle: after 1st TPR and 1st TPRx deoxygenation. All XRD were analyzed after sample cooling to room temperature and then exposure to air.

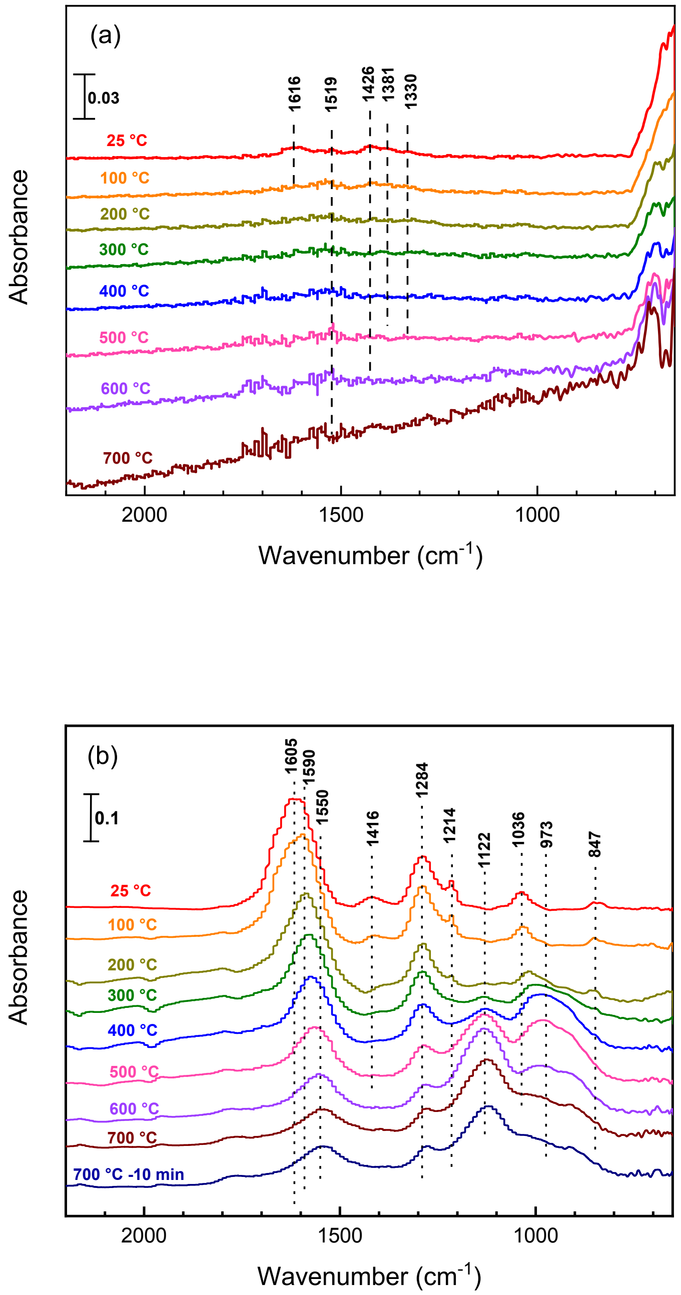

Figure S4. In situ DRIFTS analysis during CO_2-TPRx over (a) reduced In_2O_3, and (b) reduced $Sm_{0.5}Ce_{0.5}O_y$. Both samples were in line reduced by TPR up to 700 ^{o}C. The background spectra were recorded over reduced samples under Ar at the specified temperature of the spectrum.

Table S4. Assignments of absorbance bands (cm^{-1}) of in situ DRIFTS during CO_2 sTPRx over selected samples after TPR

Sample<br>Species	$In_{0.5}Ce_{0.5}O_x$	CeO_2	In_2O_3	$Sm_{0.5}Ce_{0.5}O_x$	references
**bicarbonate**	1631	1456,1630	1426,1616	847,1036, 1214,1416, 1621	CeO_2: 1021, 1218, 1414, 1619 [S1]<br>In_2O_3: 1291, 1429, 1645 [S2]<br>SmCe: 823, 1022, 1217, 1410, 1614 [S3]
**bidentate carbonate**	1280,1580	849,1032, 1282,1400, 1585,	1381, 1519	847, 1020, 1284, 1590	CeO_2: 1021, 1287, 1570 [S1]<br>In_2O_3:1394, 1510 [S2]<br>SmCe: 856, 1022, 1296, 1583 [S3]
**monodentate carbonate**	1057,1312, 1487	860,1040, 1316,1545,	1330	1020, 1122, 1284, 1550	CeO_2: 1021, 1373, 1442 [S1]<br>In_2O_3: 1338, 1594 [S2]<br>SmCe: 856, 1088, 1358, 1452 [S3]
**polydentate carbonate**		1456,1540			CeO_2: 1070, 1360, 1460, 1575 [S4,S5]

[S1] Z.-J. Gong, Y.-R. Li, H.-L. Wu, S.D. Lin, W.-Y. Yu, Direct copolymerization of carbon dioxide and 1, 4-butanediol enhanced by ceria nanorod catalyst, Appl. Catal. B: Environ., 265 (2020) 118524. 97

[S2] Y. Wang, J. Zhao, Y. Li, C. Wang, Selective photocatalytic CO_2 reduction to CH_4 over Pt/In_2O_3: Significant role of hydrogen adatom, Appl. Catal. B: Environ., 226 (2018) 544-553.

[S3] M. Grunbacher, B. Klotzer, S. Penner, CO_2 Reduction by Hydrogen Pre-Reduced Acceptor-Doped Ceria, ChemPhysChem, 20 (2019) 1706-1718.

[S4] K. Yoshikawa, H. Sato, M. Kaneeda, J.N. Kondo, Synthesis and analysis of CO_2 adsorbents based on cerium oxide, J. CO_2 Utilization, 8 (2014) 34-38.

[S5] M. Li, U. Tumuluri, Z. Wu, S. Dai, Effect of dopants on the adsorption of carbon dioxide on ceria surfaces, ChemSusChem, 8 (2015) 3651-3660.

Table S5. Calculated H_2 consumption, CO_2 consumption, and CO formation during 5 cycles of isothermal H_2-reduction-CO_2-deoxygenation over $In_{0.5}Ce_{0.5}O_x$ at 700 ^oC.

Cycle	H_2 consumption [1]	CO_2 consumption [1]	CO formation [1]
1st	2807	1008	1453
2nd	1638	1688	1777
3rd	1288	1463	1492
4th	1550	1705	1620
5th	1518	1710	1642

[1] The unit is in μmol/g for H_2 consumption during reduction and CO_2 consumption/CO formation during CO_2 deoxygenation.

Table S6. Evaluation of the extent of reduction of $In_xCe_{1-x}O_y$ during TPR (to 700 °C).

Sample	In content [1] (mmol/g)	H_2 consumption (μmol/g)		Extent of In reduction [2]
		1st TPR	2nd TPR	
$In_{0.2}Ce_{0.8}O_x$	1.20-1.21	1562	1448	0.79-0.87
$In_{0.5}Ce_{0.5}O_x$	3.14-3.21	3533	3473	0.72-0.75
In_2O_3	7.2	2608	2094	0.19-0.24

[1] The In content of $In_xCe_{1-x}O_y$ is subjected to the influence of y, i.e., the stoichiometric ratio of O. We evaluated the In content by two scenarios of stoichiometry, as (i) $In_2O_3 + CeO_2$, and (ii) $In_xCe_{1-x}O_2$. This lead to a range of In content of $In_xCe_{1-x}O_y$ as shown in the table.

[2] The extent of In reduction is calculated by assuming that all the H_2 consumption in TPR was for converting In^{3+} to In^0, with the basis of the calculated In content.
